# Provoked Mesenteric Venous Thrombosis Following Emergent Appendectomy: A Case Report

**DOI:** 10.7759/cureus.88070

**Published:** 2025-07-16

**Authors:** Muzamil Ahmad, Maya Nassif, Mark Herman

**Affiliations:** 1 Department of Surgery, Oakland University William Beaumont School of Medicine, Rochester, USA; 2 Department of Surgery, Corewell Health William Beaumont University Hospital, Royal Oak, USA

**Keywords:** anticoagulant therapy, appendectomy, laparoscopy, mesenteric venous thrombosis, oral contraceptives, thrombosis

## Abstract

Mesenteric venous thrombosis (MVT) is a rare, life-threatening condition characterized by the formation of a thrombus in the mesenteric venous system. MVT has been reported as a rare complication of laparoscopic surgery and oral contraceptive (OC) use; however, literature remains limited on a reported case highlighting the combinatorial effect of these two independent risk factors in provoking an MVT. We present the case of a 48-year-old female patient taking oral contraceptives who presented with nonspecific abdominal symptoms and unremarkable physical examination findings following an uneventful emergent laparoscopic appendectomy eleven days prior. Computed tomography (CT) angiography revealed extensive superior mesenteric venous thrombosis. No signs of bowel ischemia were present, and the patient was successfully treated with anticoagulant therapy. This report underscores the importance of clinical vigilance for mesenteric venous thrombosis in patients taking oral contraceptives who present with nonspecific abdominal pain following emergent abdominal surgery.

## Introduction

Mesenteric venous thrombosis (MVT) is the rare formation of a blood clot in the superior mesenteric vein (90% of cases) or inferior mesenteric vein (10% of cases) with an estimated annual incidence of 2.7 cases per 100,000 people [1]. MVT can be categorized as acute, subacute, or chronic, although acute disease is significant for its 30-day mortality rate approaching 30% [2]. MVT poses a risk for intestinal infarction, bowel perforation, and sepsis, and is ultimately life-threatening if not promptly treated. Underlying factors that can trigger MVT formation include hypercoagulable states such as inherited thrombophilias, as well as inflammatory bowel disease, malignancy, and stasis [3]. Notably, MVT is a rare and potentially fatal complication of laparoscopic surgery, especially in patients with underlying hypercoagulability, such as those taking an oral contraceptive (OC) [4]. The procoagulation effects of OCs are extensively documented; synthetic estrogen is linked to increased plasma fibrinogen, activation of coagulation factors, and decreased natural anticoagulants such as protein S and antithrombin [5]. MVT induced by OC use is reported to account for 4%-5% of all MVTs [6]. While there have been reports of laparoscopic surgery and OC use independently triggering MVT, the literature remains limited in reporting their combinatorial effect. This case report underscores the need to rule out MVT in post-abdominal surgery patients presenting with nonspecific abdominal pain, especially in those using OCs, as well as the importance of early diagnosis and prompt anticoagulant therapy to improve patient outcomes. This need is particularly critical in patients undergoing emergent abdominal surgery without adequate time to discontinue OC use to mitigate postoperative thrombotic events.

## Case presentation

**Figure 1:**
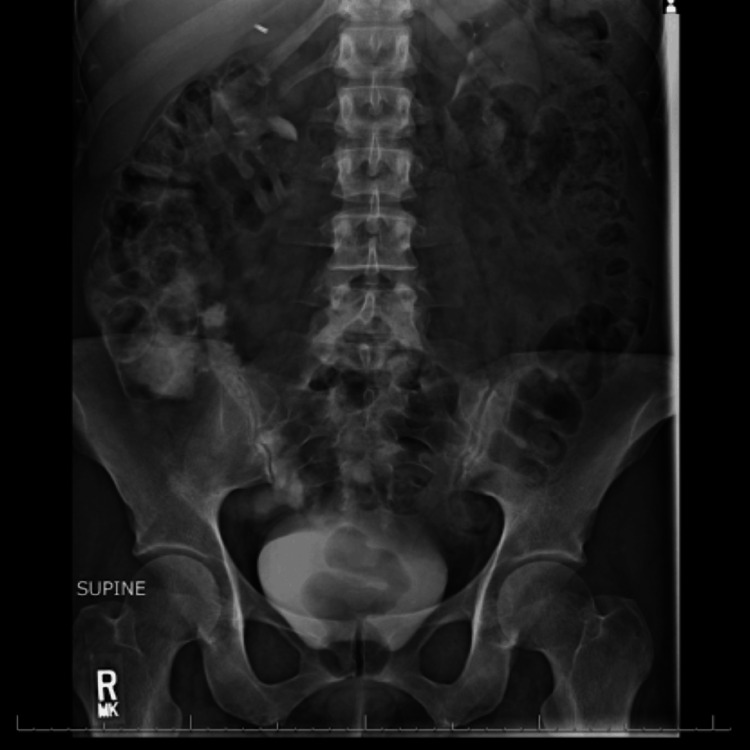
Supine abdominal radiograph showing moderate colonic stool burden

**Figure 2:**
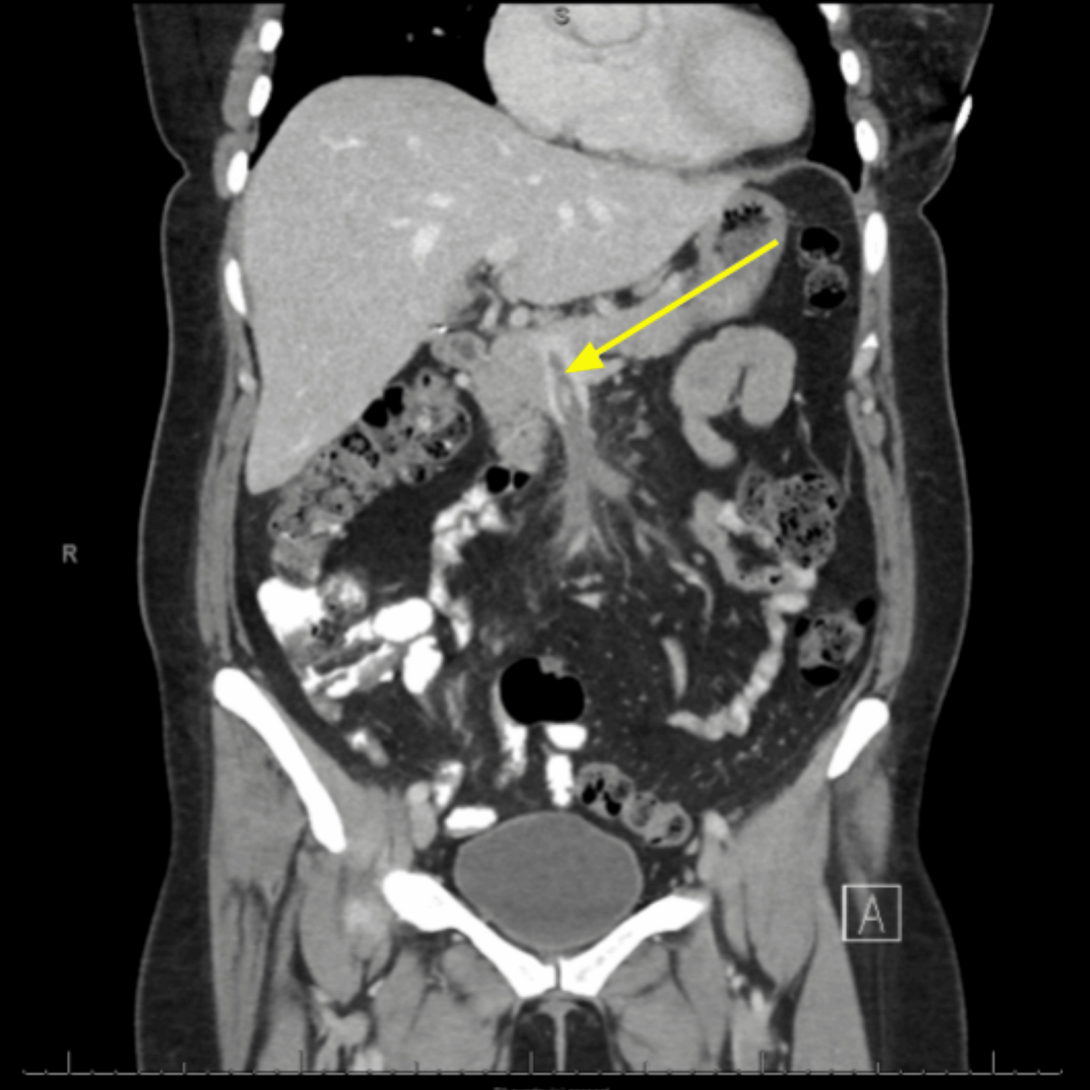
Coronal contrast-enhanced CT angiography of the abdomen revealing a non-opacified filling defect in the superior mesenteric vein extending into the portal venous confluence (yellow arrow) CT: computed tomography

**Figure 3:**
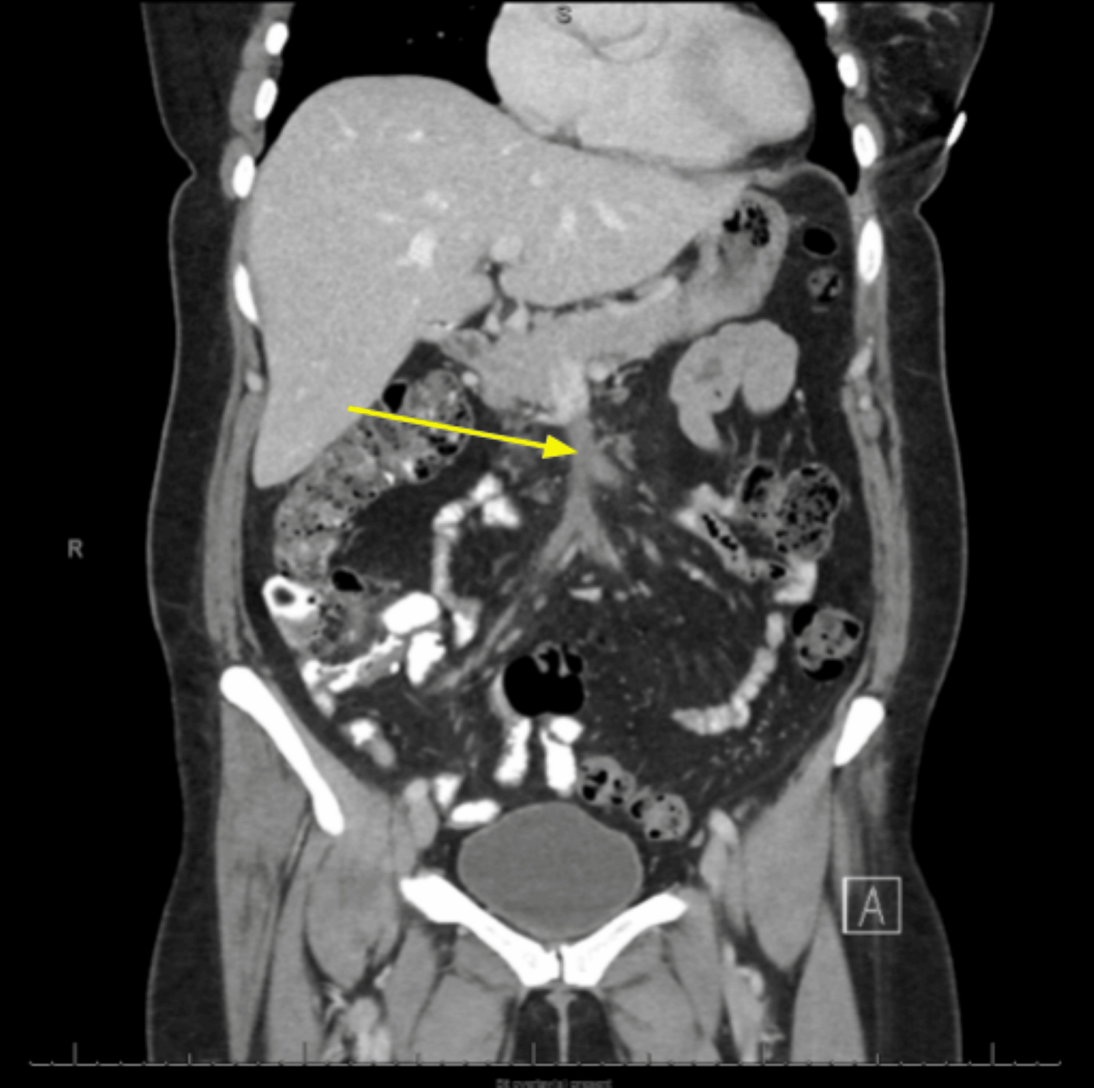
Coronal contrast-enhanced CT angiography of the abdomen revealing a non-opacified filling defect in the superior mesenteric vein (yellow arrow) CT: computed tomography

**Figure 4:**
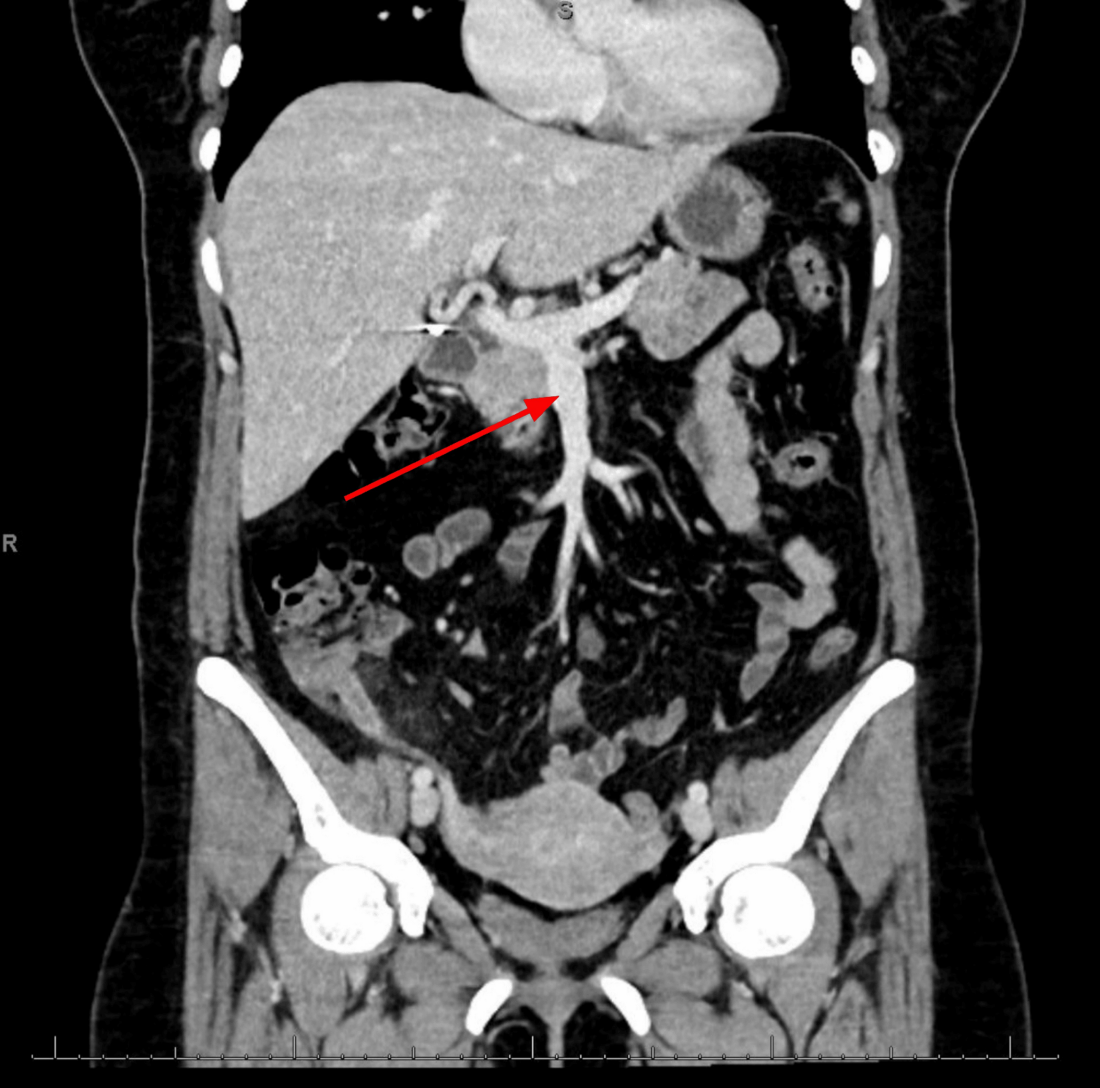
Coronal CT of the abdomen taken immediately before the patient's appendectomy, demonstrating no evidence of thrombus within the mesenteric veins (red arrow) CT: computed tomography

A 48-year-old woman on a combined oral contraceptive (OC) (ethinylestradiol + levonorgestrel) underwent an uneventful emergent laparoscopic appendectomy for appendicitis and was discharged home the same day following an unremarkable postoperative course. On postoperative day 11, she presented to the emergency department with nonspecific abdominal pain associated with two days of constipation and nausea. On admission, her blood pressure, heart rate, and respirations were within normal limits. Physical examination revealed generalized abdominal tenderness without rebound tenderness, rigidity, or guarding. The patient was hemodynamically stable, and laboratory tests (comprehensive metabolic panel, complete blood count, lipase, lactic acid, and ꞵ-human chorionic gonadotropin) were unremarkable (Table 1). Plain abdominal radiography revealed moderate colonic stool burden without evidence of intra-abdominal free air, bowel obstruction, dilated bowel loops, or differential air-fluid levels (Figure 1). Computed tomography (CT) of the abdomen, with intravenous (IV) and oral contrast, revealed extensive intraluminal thrombosis extending from the superior mesenteric vein to the portal confluence, with filling defects and mildly thickened small bowel loops (Figures 2, 3). These findings were markedly different from those observed on the patient's abdominal CT scan obtained 11 days earlier, immediately prior to the appendectomy (Figure 4).

**Table 1:** Pertinent laboratory values on presentation WBC: white blood cell, CO2: carbon dioxide, BUN: blood urea nitrogen, AST: aspartate aminotransferase, ALT: alanine aminotransferase, ꞵ-hCG: ꞵ-human chorionic gonadotropin

Test	Result	Reference range
WBC	9.1 × 10^9^/L	3.3-10.7 × 10^9^/L
Hemoglobin	12.9 g/dL	12.1-15.0 g/dL
Hematocrit	40.3%	35.4%-44.2%
Platelets	251 × 10^9^/L	150-400 × 10^9^/L
Neutrophils	5.6 × 10^9^/L	1.6-7.2 × 10^9^/L
CO2 (bicarbonate)	23 mmol/L	20-29 mmol/L
Anion gap	8	5-17
BUN	10 mg/dL	7-25 mg/dL
Creatinine	0.85 mg/dL	0.50-1.10 mg/dL
AST	14 U/L	<35 U/L
ALT	19 U/L	8-37 U/L
Bilirubin (total)	0.3 mg/dL	0.3-1.2 mg/dL
Lipase	19 U/L	7-60 U/L
Lactic acid	0.6 mmol/L	0.5-2.0 mmol/L
ꞵ-hCG	<1 mIU/mL	0-5 mIU/mL

A diagnosis of extensive acute mesenteric venous thrombosis was made with no signs of mesenteric ischemia or infarction. Immediate intravenous (IV) heparin therapy was initiated for 48 hours, and the patient was admitted to closely monitor for signs of mesenteric ischemia. The patient remained stable, without signs of bowel ischemia or bleeding, and had a return of regular bowel movements three days after heparin initiation. Following IV anticoagulant therapy, the patient was transitioned to apixaban, a direct oral anticoagulant (DOAC), for a minimum of six months. The OC was discontinued, and the patient was discharged with plans for an abdominal magnetic resonance imaging (MRI) and hematology follow-up. Hematology consultation recommended against a hypercoagulability workup due to the high likelihood that the thrombosis was provoked by OC use and recent laparoscopic surgery. At a follow-up appointment one month post-discharge, the patient remained asymptomatic and without signs suggestive of mesenteric ischemia.

## Discussion

Mesenteric venous thrombosis is a rare condition that may rapidly progress to life-threatening mesenteric ischemia, bowel infarction, or death. With an incidence of 2.7 per 100,000 people, MVT accounts for approximately 5%-15% of all acute mesenteric ischemia cases [1,3]. Patients are typically between 40 and 60 years old, with no significant difference in sex distribution; however, patients can be of any age, including pediatric patients with prothrombotic conditions [3]. MVT formation may be idiopathic, but is most often associated with underlying hypercoagulability or trauma. Unlike mesenteric arterial ischemia, the clinical course of MVT is more insidious with nonspecific findings. The most common vessel involved is the superior mesenteric vein, which can extend into the portal or splenic veins. With early diagnosis and intervention, the prognosis of MVT improves, preventing or limiting bowel ischemia.

Patients with MVT most often present with unremarkable physical examination findings and nonspecific symptoms, which may delay diagnosis. The most frequent presenting symptom is diffuse abdominal pain that is out of proportion to physical examination findings. Additionally, patients may present with nausea, vomiting, constipation, diarrhea, or fever [7]. As the MVT progresses, the thrombosis may extend into the venous arcades and vasa recta, with complete occlusion of venous return leading to rapid bowel infarction. In these cases, patients may have signs of peritonitis, bowel infarction, ascites, or sepsis, indicating transmural ischemia or necrosis of the bowel [1].

Risk factors for developing an MVT include local abdominal inflammatory states, malignancy, trauma, stasis, hypercoagulability, or recent abdominal surgery [1,8]. Local abdominal inflammatory pathologies such as diverticulitis and appendicitis notably demonstrate a higher risk for postoperative MVT [4]. Systemic risk factors include inherited thrombophilias such as factor V Leiden, prothrombin 20210A, protein C/S deficiency, antithrombin III deficiency, antiphospholipid syndrome, and JAK2-mutated myeloproliferative disorders [1,8]. Previously published cases have reported the rare association between laparoscopic surgery and MVT. In the study by Kim et al., 0.3% of patients who underwent laparoscopic bariatric surgery developed a portomesenteric venous thrombosis [9]. It is estimated that 44% of patients who developed a postoperative MVT had an undiagnosed hypercoagulable state. Similarly, rare associations between OC use and MVT have been reported. Zhao et al. reported that approximately 4%-5% of all MVTs are provoked by the use of OCs due to the prothrombotic effect of estrogen [6]. Due to this thrombotic risk, OCs are typically discontinued 4-6 weeks prior to surgery [10]; however, this is not feasible in emergent cases, increasing the likelihood of postoperative thrombotic events.

Diagnosing an MVT requires a high degree of clinical suspicion, especially in patients with diffuse abdominal pain and risk factors associated with MVT. This becomes increasingly crucial in patients with no previous history of thrombotic diseases and no family history of thrombophilia, such as in this case, where thrombosis may not be obvious. CT angiography of the abdomen is the gold standard for diagnosis [1]. The most common CT angiography findings indicative of MVT include a thrombus within the mesenteric veins, circumferential mesenteric wall thickening, mesenteric edema, and venous filling defects [11]. Laboratory studies are nonspecific but may be remarkable for elevated D-dimer, elevated lactate, leukocytosis, or metabolic acidosis [12,13]. If signs of bowel necrosis are present, exploratory laparotomy may be required.

Systemic anticoagulation with heparin should be initiated immediately after the diagnosis of MVT is made, unless contraindicated. OCs must be discontinued. Patients without signs of peritonitis or bowel necrosis can be managed conservatively with anticoagulant therapy and close medical monitoring. If signs of peritonitis or bowel infarction are present, prompt surgical intervention is necessary. After stabilization, patients are transitioned to long-term anticoagulant therapy, such as DOACs or warfarin, for at least 3-6 months or indefinitely if a hypercoagulable state is present [14].

Patient prognosis largely depends on how quickly a diagnosis is made and treatment is initiated. The 30-day mortality rate of an acute MVT is approximately 32.1% [2]. With rapid diagnosis and intervention, patient mortality can be reduced to less than 10%. If treatment is delayed by just 6-12 hours, the mortality rate increases to 50%-60% [15].

In this particular case, the combined effect of OC use and recent laparoscopic surgery for an inflammatory pathology placed this patient at an increased risk for developing an MVT. The absence of bowel ischemia allowed for conservative treatment with heparin therapy without requiring surgical intervention. Because there is no opportunity for preoperative discontinuation of OCs ahead of emergency abdominal surgeries, this report stresses the importance of perioperative risk mitigation for this rare pathology in susceptible patients. Given the routine use of OCs and laparoscopic surgery, further research should seek to quantify the risk for MVT formation in patients using OCs after emergent and non-emergent laparoscopic abdominal surgery.

## Conclusions

This case report demonstrates the importance of maintaining a high index of clinical suspicion in order to achieve early diagnosis and treatment of mesenteric venous thrombosis. In this report, the nonspecific abdominal pain after recent abdominal surgery and concurrent use of OCs warranted prompt imaging studies for further evaluation. A CT angiography of the abdomen was utilized to make a definitive diagnosis of mesenteric venous thrombosis. Because the imaging studies were unremarkable for signs of bowel necrosis or infarction, the patient was managed conservatively with anticoagulant therapy. Given the nonspecific presentation yet life-threatening nature of MVTs, a comprehensive patient assessment for identifiable risk factors and early identification are essential to improving patient survival and long-term outcomes.
